# Author Reply to “Reference Paper Maroun et al. Endoscopic discectomy for L4-L5 disc herniation a meta-analysis comparing transforaminal and interlaminar approaches”

**DOI:** 10.1016/j.bas.2026.106041

**Published:** 2026-04-08

**Authors:** Youssef Jamaleddine, Ralph Maroun, Mohammad Badra

**Affiliations:** a Department of Orthopedic Surgery, Lebanese American University Medical Center-Rizk Hospital, Lebanon; b Department of Orthopedic Surgery, Lebanese University, Beirut, Lebanon; c Department of Orthopedics and Traumatology, Clemenceau Medical Center, Lebanon; d Department of Orthopedic Surgery, Faculty of Medicine, Balamand University, Lebanon

Dear Editor,

We thank Pongmanee and Kim for their interest in our meta-analysis (Maroun et al., 2026) and for their thoughtful comments (Pongmanee and Kim, 2026) regarding our work. We agree with the important general principle emphasized in their letter, namely that approach selection in endoscopic lumbar discectomy should be informed by pathology location, migration pattern, and patient-specific anatomy. This concept is fully consistent with our own discussion and conclusion, in which we stated that the choice between transforaminal and interlaminar approaches should be guided by surgeon experience, patient-specific anatomy, and resource availability (Maroun et al., 2026).

At the same time, we would like to clarify the scope of our conclusion. Our meta-analysis was designed to answer a level-specific comparative question at L4-L5, namely whether the currently available comparative literature demonstrates clear overall superiority of one approach over the other across the principal reported outcomes. Based on six studies including 456 patients, no significant between-group differences were identified for overall complications, reoperation, operative time, length of stay, ODI improvement, or pain improvement (Maroun et al., 2026). Accordingly, our conclusion should not be interpreted as claiming universal interchangeability of the two approaches in every anatomical scenario, but rather that the currently available aggregate L4-L5 evidence does not demonstrate a consistent overall advantage of one approach over the other (Maroun et al., 2026).

We agree that the marked heterogeneity in operative time warrants cautious interpretation, as noted in the letter (Pongmanee and Kim, 2026). In particular, Huang et al. evaluated highly down-migrated L4-5 disc herniations and reported shorter operative and fluoroscopy times with the interlaminar approach, suggesting a technical advantage of IL in that specific morphology (Huang et al., 2021). However, we respectfully do not believe that this observation fundamentally overturns the implications of our pooled findings. Operative-time heterogeneity is likely multifactorial and may reflect not only disc morphology and migration, but also surgeon learning curve, case selection, technical variation, and perioperative workflow (Maroun et al., 2026).

Importantly, the available L4-L5 comparative literature itself supports a more nuanced interpretation that remains fully compatible with our findings. Several level-specific studies have shown that both transforaminal and interlaminar approaches can be used within selected L4-L5 pathology subgroups, although their relative technical advantages may differ according to morphology. Huang et al. compared the two approaches in highly down-migrated L4-L5 herniation (Huang et al., 2021). Pruttikul et al. compared them in central-paracentral L4-L5 disc herniation and found similar clinical outcomes between approaches (Pruttikul et al., 2025). Takebayashi et al. compared them in L4-L5 lumbar disc herniation and concluded that both approaches could be safely and effectively performed, while approach selection should be based on patient-specific anatomical characteristics and preoperative radiological findings (Takebayashi et al., 2023). Thus, the most accurate interpretation is not that the two approaches are universally interchangeable, but that within the currently available L4-L5 literature, both can achieve favorable outcomes when applied to appropriately selected anatomical scenarios.

We also appreciate the authors’ reference to anatomy-based decision frameworks (Pongmanee and Kim, 2026). The algorithm proposed by Kotheeranurak et al. provides a useful conceptual framework for approach selection (Kotheeranurak et al., 2023). In our view, however, such an algorithm should be regarded as complementary context for interpretation rather than as a basis for overturning the central result of our meta-analysis. Our findings are fully compatible with anatomy-guided decision-making, since they indicate that when patients are appropriately selected, both approaches can produce favorable outcomes, while the currently pooled comparative evidence at L4-L5 does not support a universal claim of superiority for one approach across the major outcomes studied (Maroun et al., 2026).

We further agree that future comparative studies and future meta-analyses would be strengthened by more consistent stratification according to disc location, migration grade, surgeon experience, and anesthetic protocol. Indeed, this limitation was already acknowledged in our article, where inconsistent reporting and the limited number of available studies prevented robust subgroup analyses (Maroun et al., 2026).

We thank Pongmanee and Kim again for their constructive comments (Pongmanee and Kim, 2026). In our view, their anatomy-based interpretation is complementary to, rather than corrective of, our principal conclusion. Our meta-analysis did not deny the importance of anatomy-guided selection. Rather, it showed that when the currently available comparative L4-L5 evidence is pooled, neither transforaminal nor interlaminar endoscopic discectomy demonstrates clear overall superiority across the principal clinical outcomes studied (Maroun et al., 2026).

## Declaration of interests

The authors declare that they have no known competing financial interests or personal relationships that could have appeared to influence the work reported in this paper.
